# Hydrogen Dynamics in *Trichodesmium* Colonies and Their Potential Role in Mineral Iron Acquisition

**DOI:** 10.3389/fmicb.2019.01565

**Published:** 2019-07-10

**Authors:** Meri Eichner, Subhajit Basu, Martha Gledhill, Dirk de Beer, Yeala Shaked

**Affiliations:** ^1^Microsensor Group, Max Planck Institute for Marine Microbiology, Bremen, Germany; ^2^The Freddy & Nadine Herrmann Institute of Earth Sciences, The Hebrew University of Jerusalem, Jerusalem, Israel; ^3^The Interuniversity Institute for Marine Sciences in Eilat, Eilat, Israel; ^4^GEOMAR Helmholtz Center for Ocean Research Kiel, Kiel, Germany

**Keywords:** *Trichodesmium*, colony, N_2_ fixation, H_2_ evolution, uptake hydrogenase, O_2_ fluxes, iron acquisition, dust

## Abstract

N_2_-fixing cyanobacteria mediate H_2_ fluxes through the opposing processes of H_2_ evolution, which is a by-product of the N_2_ fixation reaction, and H_2_ uptake, which is driven by uptake hydrogenases. Here, we used microelectrodes to characterize H_2_ and O_2_ dynamics in single natural colonies of the globally important N_2_ fixer *Trichodesmium* collected from the Gulf of Eilat. We observed gradually changing H_2_ dynamics over the course of the day, including both net H_2_ evolution and net H_2_ uptake, as well as large differences in H_2_ fluxes between individual colonies. Net H_2_ uptake was observed in colonies amended with H_2_ in both light and dark. Net H_2_ evolution was recorded in the light only, reflecting light-dependent N_2_ fixation coupled to H_2_ evolution. Both net H_2_ evolution and H_2_ uptake rates were higher before 2 pm than later in the day. These pronounced H_2_ dynamics in the morning coincided with strong net O_2_ uptake and the previously reported diel peak in N_2_ fixation. Later in the afternoon, when photosynthesis rates determined by O_2_ measurements were highest, and N_2_ fixation rates decrease according to previous studies, the H_2_ dynamics were also less pronounced. Thus, the observed diel variations in H_2_ dynamics reflect diel changes in the rates of O_2_ consumption and N_2_ fixation. Remarkably, the presence of H_2_ strongly stimulated the uptake of mineral iron by natural colonies. The magnitude of this effect was dependent on the time of day, with the strongest response in incubations that started before 2 pm, i.e., the period that covered the time of highest uptake hydrogenase activity. Based on these findings, we propose that by providing an electron source for mineral iron reduction in N_2_-fixing cells, H_2_ may contribute to iron uptake in *Trichodesmium* colonies.

## Introduction

Marine primary productivity is often limited by the availability of dissolved organic or inorganic nitrogen (Moore et al., 2013). Diazotrophic cyanobacteria can access an additional nitrogen source, dinitrogen (N_2_) gas, and reduce it to ammonia, thereby making it available for other phytoplankton. As part of the N_2_ fixation reaction catalyzed by nitrogenase, an equimolar amount of hydrogen (H_2_) is produced:

(1) $${\text{N}}_{2}+8{\text{e}}^{-}+8{\text{H}}^{+}+16\text{ATP}\to 2{\text{NH}}_{3}+{\text{H}}_{2}+16(\text{ADP}+{\text{P}}_{i})$$

Since the reduction of protons (H^+^) consumes both reducing equivalents and ATP, H_2_ evolution contributes to the high energy costs related to N_2_ fixation. However, all N_2_-fixing cyanobacteria analyzed so far have either uptake hydrogenases or bidirectional hydrogenases that allow them to recycle some of this H_2_ (Tamagnini et al., 2002). As these hydrogenases can feed electrons from H_2_ into the respiratory electron transport chain, the uptake of H_2_ provides a mechanism for recycling reducing equivalents.

The cyanobacterium *Trichodesmium* is one of the major marine N_2_ fixers and has been estimated to contribute up to 50% to total nitrogen fixation in some areas (e.g., Karl et al., 1997; Dore et al., 2002). A range of different protective mechanisms has been proposed to shield the O_2_-sensitive nitrogenase from photosynthetically evolved O_2_ in *Trichodesmium.* Firstly, spatial separation of N_2_ fixation and photosynthesis was suggested for the colony-level (Paerl and Bebout, 1988) as well as the single-cell level (N_2_-fixing cells termed diazocytes; Bergman and Carpenter, 1991; Berman-Frank et al., 2001). Additionally, nitrogenase activity was shown to be strictly regulated over the diel cycle by daily synthesis of the nitrogenase pool in the morning and inactivation by post-translational modification in the afternoon, allowing for a relatively narrow peak of activity around midday (Zehr et al., 1993; Berman-Frank et al., 2001). A down-regulation of photosynthesis during this midday peak in N_2_ fixation was suggested as a mechanism to protect nitrogenase from O_2_ (Berman-Frank et al., 2001). Regarding the H_2_ metabolism of *Trichodesmium*, the genome of *Trichodesmium erythraeum* IMS101 encodes for the uptake hydrogenase *hupSL*^1^ and previous studies on its H_2_ metabolism showed that it can recapture ca. 70% of the evolved H_2_ (Wilson et al., 2010, 2012).

In natural systems, *Trichodesmium* often forms colonies that host a wide range of epibiotic bacteria with diverse functions (Hewson et al., 2009; Hmelo et al., 2012; Lee et al., 2017; Frischkorn et al., 2018). Apart from providing a substrate for bacteria, colonies can induce formation of distinct chemical microenvironments. Early studies suggested anoxic microenvironments in *Trichodesmium* colonies to facilitate N_2_ fixation in colonies (Paerl and Bebout, 1988), potentially including the Knallgas reaction (i.e., the reaction of H_2_ with O_2_ to form H_2_O) as a mechanism to reduce O_2_ concentrations (Saino and Hattori, 1982). However, more recent studies showed that during day-time in the light, when photosynthesis is active, O_2_ concentrations in colonies can increase to as much as 200% of air saturation, which would impose a considerable challenge to the O_2_-sensitive nitrogenase in *Trichodesmium* (Eichner et al., 2017, 2019). In view of this colony-specific potential impediment to N_2_ fixation, it becomes all the more relevant to identify the role of colony formation.

Recent studies have suggested colony formation to facilitate iron acquisition from dust, as *Trichodesmium* colonies were shown to efficiently capture dust particles, move them to the colony center and enhance iron dissolution from the dust (Rubin et al., 2011). The physiological and biochemical mechanisms of dust particle translocation to the colony center and iron dissolution from dust by *Trichodesmium* are still under investigation. In a parallel study (Eichner et al., submitted), we examined whether chemical gradients within colonies provide beneficial conditions for iron dissolution in the colony center. Measuring O_2_ and pH gradients within *Trichodesmium* colonies with microsensors, we found that the respiration-induced decreases in O_2_ and pH in colonies are not large enough to significantly enhance iron dissolution. Hence, the active centering of dust particles in the colony must have a different reason.

The release of soluble iron from minerals, where it is generally present in the form of Fe(III) oxide or oxyhydroxide, can be facilitated by ligand (L) binding (Eq. 2) and/or the reduction of Fe^3+^ to Fe^2+^ (Eq. 3):

(2) $$\text{Fe}{\left(\text{OH}\right)}_{3(\text{solid})}+{\text{L}}^{-}+3{\text{H}}^{+}\to {\left[\text{FeL}\right]}_{\left(\text{soluble}\right)}+3{\text{H}}_{2}\text{O}$$

(3) $$\text{Fe}{\left(\text{OH}\right)}_{3(\text{solid})}+{\text{e}}^{-}+3{\text{H}}^{+}\to {\text{Fe}}^{2+}{}_{\left(\text{soluble}\right)}+3{\text{H}}_{2}\text{O}$$

Mineral iron dissolution by *Trichodesmium* has been suggested to involve ligand-promoted dissolution (Basu et al., in press) and/or reductive dissolution (Rubin et al., 2011). While *Trichodesmium* has been demonstrated to reduce dissolved iron (Roe and Barbeau, 2014; Lis et al., 2015), direct measurements of mineral iron reduction by this organism are still lacking. Reductive dissolution of mineral iron most likely occurs in the vicinity of the cells in the colony center, where the minerals are concentrated. One possibility is electron transfer to Fe(III) directly on the cell surface, using electrons derived from photosynthesis or respiration, by a yet unidentified mechanism. Alternatively, accumulation of a reductant in the colony microenvironment might enable reductive dissolution. Here, we propose that the uptake of H_2_, which is produced during N_2_ fixation, might play a previously unrecognized role in supplying reducing power for iron acquisition. Specifically, we hypothesized that H_2_ can act as a reductant for mineral-bound Fe(III), which is then liberated from the dust matrix for uptake and assimilation.

To address this hypothesis, we firstly used microelectrodes to characterize H_2_ fluxes and their dynamics in relation to O_2_ fluxes over the diel cycle in the microenvironment of single colonies collected in the Gulf of Eilat. In a second step, we used this data to analyze the kinetics of H_2_ uptake under different light conditions and stages of the diel cycle. Finally, to evaluate the potential of H_2_ as a reductant for mineral iron dissolution and acquisition, we investigated whether addition of H_2_ affects the uptake of mineral iron by *Trichodesmium* colonies.

## Materials and Methods

### Colony Sampling

*Trichodesmium* colonies were sampled from the Gulf of Eilat/Aqaba in the Red Sea over a period of 2 months from March to May 2018. Colonies were collected with a 200 μm net, either placed statically at ca. 1–2 m depth on a pole extended from the pier (3–4 m bottom depth) for approx. 2 h, or by vertical net tows from 20 m to the surface carried out from a boat at ca. 300 m bottom depth in the Gulf. Puff-shaped colonies were then hand-picked with Pasteur pipettes and washed with trace metal free filtered sea water (cleaned using ion exchange resin Chelex 100). Colonies were collected multiple times during the day and kept in filtered seawater under stable light conditions (ca. 130 μmol photons m^−2^ s^−1^) at 25°C for ca. 1–5 h until measurements were started. Assuming that the diel rhythm of metabolic activity by the colonies is maintained under these conditions, rate measurements over the diel cycle are reported relative to the time of measurement rather than sample collection time.

### Microsensor Measurements

Microsensor measurements were performed on single colonies. The colonies were placed in filtered seawater in Petri dishes of approx. 50 ml volume, and held in position with a thin glass needle above a nylon mesh to ensure unperturbed diffusion in all directions. The seawater was slowly mixed during measurements by an air flow over the water surface produced with a pipette connected to an aquarium air pump. Measurements were performed at approx. 25°C and 350–450 μmol photons m^−2^ s^−1^, unless specified otherwise.

Clark-type O_2_ microelectrodes with ca. 10 μm tip diameter were made at Max Planck Institute for Marine Microbiology as described earlier (e.g., Kühl and Revsbech, 2000). O_2_ sensors were calibrated with seawater bubbled with N_2_ gas (0% reading) and with air-equilibrated seawater (21% reading; 212 μmol L^−1^ O_2_ at 23°C and salinity of 40) and corrected for changes in electrode performance over the course of the experiments by assuming that O_2_ concentrations in the bulk seawater surrounding the colonies (i.e., outside of the microenvironment affected by cellular O_2_ fluxes) was in equilibrium with the room air, which was facilitated by the large surface area of the Petri dish used for measurements. H_2_ microelectrodes (Unisense) had a tip size of 50 μm and minimum detection limit below 50 nmol L^−1^ and were calibrated with solutions of known H_2_ concentrations prepared by dilution of H_2_-saturated seawater (solubility of 652 μmol L^−1^ at 26°C and salinity of 40) assuming a linear response of the electrode. Calibrations were performed daily to account for changes in electrode sensitivity over the duration of the study.

**FIGURE 1:**
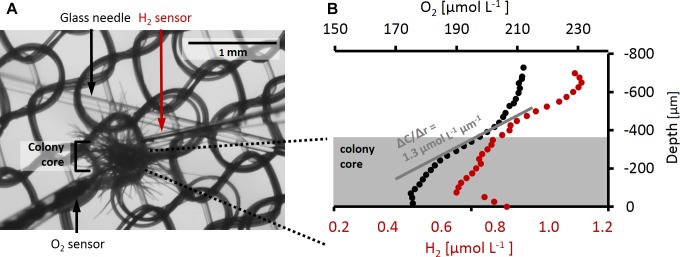
**(A)** *Trichodesmium* colony during a microsensor measurement. O_2_ sensor and H_2_ sensor as well as the glass needle and nylon mesh holding the colony in place are shown. The size of the colony core as used for flux calculations is indicated by the bracket. **(B)** Example of O_2_ and H_2_ depth profiles measured simultaneously on the same colony. Measurements were performed in the dark after H_2_ addition. Gray shading indicates the approximate area taken by the colony core, the center of the colony is approximately at depth –70 μm. The gray line shows an example of a linear fit to the O_2_ gradient at the colony surface. The slope of this line gives ΔC/Δr which is used to calculate the flux of O_2_ across the colony surface according to Eq. 4.

For recording depth profiles, microelectrodes were moved toward and through the colonies at 50–100 μm step size with a micromanipulator (VT-80, Micos) driven by a motor (Faulhaber minimotor, SA) while observing the colony at ca. 5× magnification in a stereomicroscope (SMZ1500, Nikon with DMC G5, Panasonic camera). For simultaneous measurements of O_2_ and H_2_ profiles on the same colony, the two sensors were mounted in a 90° angle (Figure 1A) on two separate micromanipulators. Each colony was photographed for determination of colony dimensions and calculation of volume and surface area assuming spherical geometry.

Net O_2_ and H_2_ fluxes in and out of the colonies were calculated from the steady state gradients at the colony surface (Figure 1B) according to Fick’s first law of diffusion:

(4) J=−D(ΔC/Δr)

where J represents the interfacial O_2_ or H_2_ flux, D the diffusion coefficient (2.2 × 10^−9^ m^2^ s^−1^ for O_2_ and 4.3 × 10^−9^ m^2^ s^−1^ for H_2_ at 25°C and salinity 40; Broecker and Peng, 1974), and ΔC the concentration difference measured over the respective distance, Δr, at the colony surface. Interfacial flux was converted to the volume-normalized rate using estimates of surface area and volume for each colony. The surface of the colony was defined visually based on measured profiles as the depth where gradients in O_2_ concentrations were steepest. Note that this was typically the boundary of the relatively dense colony core (as indicated in Figure 1), i.e., excluding single filaments protruding outward from the core area.

For detecting not only net H_2_ evolution but also potential net H_2_ uptake rates, H_2_ profiles were recorded under artificially elevated H_2_ concentrations. To this end, H_2_ was added by pipetting H_2_-saturated seawater into the measurement container, reaching final concentrations from below detection limit up to 13 μmol L^−1^ (6 ± 12 μmol L^−1^).

### Iron Uptake Measurements

Iron uptake from radioactively labeled ^55^ferrihydrite colloids by natural colonies was determined as described by Basu and Shaked (2018). Briefly, ca. 50 colonies were placed in 5 ml acid-cleaned glass vials and ^55^ferrihydrite was added to a final concentration of 100 nmol L^−1^. Vials were then amended with H_2_-saturated seawater to reach a final H_2_ concentration of 45 ± 34 μmol L^−1^ (18–102 μmol L^−1^), closed without headspace and incubated at ca. 300 μmol photons m^−2^ s^−1^ and 25°C for 3–12 h. Control samples without added H_2_ were prepared for each experiment and H_2_ concentrations in all vials were determined at the end of incubations with H_2_ microelectrodes. At the end of the incubations, samples were transferred into Ti-EDTA-citrate solution (20 min) to ensure removal of absorbed ^55^ferrihydrite and effective detachment of bacteria, and subsequently filtered on 8 μm polycarbonate filters to retain *Trichodesmium* biomass. ^55^Fe internalized by *Trichodesmium* was determined by β-counting of the filters in a Tri-carb 1600 CA liquid scintillation counter (Packard).

## Results

### H_2_ Dynamics Over the Diel Cycle – Relation to O_2_ Fluxes

**FIGURE 2:**
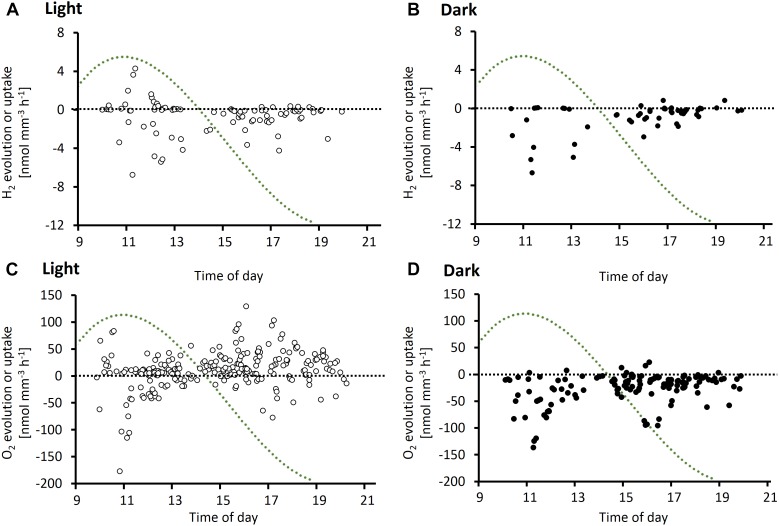
Diel cycle of potential H_2_ evolution or uptake rates **(A,B)** and O_2_ evolution or uptake rates **(C,D)** based on depth profiles measured on *Trichodesmium* colonies in the light **(A,C)** and dark **(B,D)**. Data points represent single measurements of H_2_ or O_2_ profiles. Measurements were performed on a total of 130 and 23 colonies for O_2_ and H_2_, respectively. *X*-axis denotes time of measurement. Note that all data shown are net rates, with positive numbers reflecting net evolution of H_2_ or O_2_ by a specific colony, and negative numbers reflecting net uptake of H_2_ or O_2_, respectively. The green dotted line represents a typical diel cycle in N_2_ fixation (arbitrary units), which was obtained by a third order polynomial fit to an average of N_2_ fixation rates measured by Berman-Frank et al. (2001), Milligan et al. (2007), Kranz et al. (2010), and Eichner et al. (2014). N_2_ fixation rates were normalized to the diel maximum in the respective study. For laboratory studies, start and end of the 12 h light period were aligned to the times of sunrise (6 am) and sunset (7 pm), respectively, in Eilat at the time of the experiments.

Microsensor measurements revealed a large variability in H_2_ and O_2_ fluxes between single colonies of *Trichodesmium* analyzed under similar conditions at the same time of the day (Figure 2). Both net evolution and net uptake of H_2_ were observed in the presence of added H_2_ (Figure 2A,B). The range of net H_2_ and O_2_ fluxes was clearly dependent on light and showed a distinct pattern over the diel cycle. Net H_2_ uptake was observed in both light and dark, whereas net H_2_ evolution was observed only in the light. Both net H_2_ evolution and net H_2_ uptake were highest during morning and midday. In the afternoon, there was no net H_2_ evolution and H_2_ uptake rates were relatively low. The peak in net H_2_ evolution and uptake in the late morning/midday coincided with a peak in O_2_ uptake (respiration) rates (Figure 2C,D) as well as the previously reported daily maximum in N_2_ fixation rates in *Trichodesmium*, ca. 5 h after beginning of the light period (e.g., Berman-Frank et al., 2001; Levitan et al., 2007; Milligan et al., 2007; Kranz et al., 2010; Eichner et al., 2014). Following a transient decrease in O_2_ fluxes (Figure 2C,D) after midday, respiration and photosynthesis increased again. Photosynthesis rates reached their daily maximum in the late afternoon, when there was no net H_2_ evolution, moderate H_2_ uptake and N_2_ fixation rates were reported to be low (Figure 2C; Berman-Frank et al., 2001; Levitan et al., 2007; Milligan et al., 2007; Kranz et al., 2010; Eichner et al., 2014).

**FIGURE 3:**
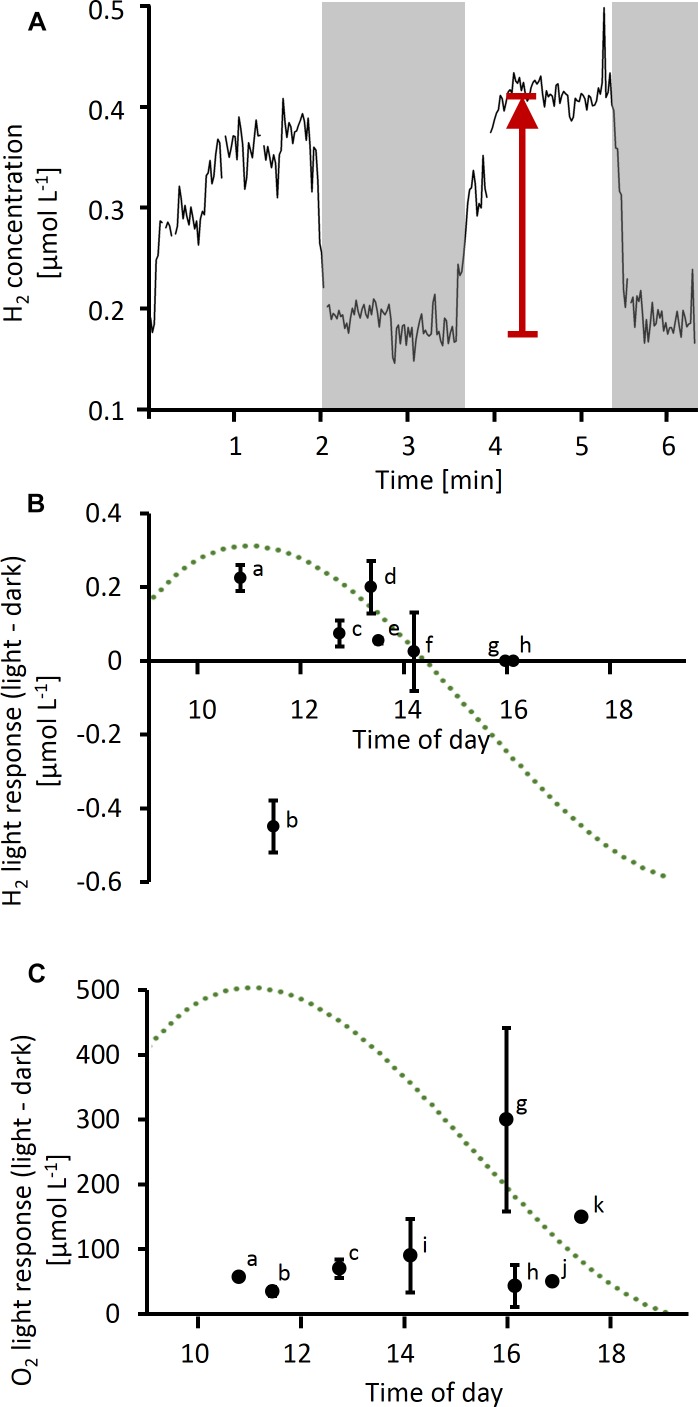
**(A)** Example of time series measurement of H_2_ performed on a single *Trichodesmium* colony. Gray shading indicates dark phases, arrow indicates light response (as plotted in **B**). **(B,C)** Light response in H_2_ **(B)** and O_2_ **(C)** concentrations (i.e., difference in concentration in light versus dark) measured at different times of the day. Data points labeled with the same letter in **(B)** and **(C)** were measured simultaneously using two microelectrodes (H_2_ and O_2_) on the same colony. Error bars indicate standard deviation of multiple measurements on the same colony (*n* ≥ 2, except for colonies j and k). The dotted line represents a typical diel cycle in N_2_ fixation (arbitrary units), which was obtained by a third order polynomial fit to N_2_ fixation rates measured in previous studies as specified in the legend of Figure 2.

In a separate experiment, O_2_ and H_2_ concentrations were followed over time at the same position close to the center of a colony during consecutive light and dark phases (Figure 3A). An increase in H_2_ concentrations by up to 200 nmol L^−1^ was observed when the lights were switched on in four out of eight colonies tested (Figure 3B). One colony tested in the morning showed the opposite response to light, i.e., higher H_2_ concentrations in the dark than in the light (colony b, Figure 3B). The response to light was strongest around midday, as observed for absolute fluxes of H_2_ (Figure 2), and in concert with the previously reported peak in N_2_ fixation. Two colonies tested in the afternoon, i.e., outside of the N_2_ fixation period, showed no light response in H_2_ fluxes, although the same colonies were clearly active as indicated by their strong response in O_2_ concentrations to light (colonies g and h, Figure 3B,C). O_2_ responses to light showed an opposite diel pattern compared to H_2_ responses, with stronger responses in the afternoon than at morning/midday (Figure 3C).

**FIGURE 4:**
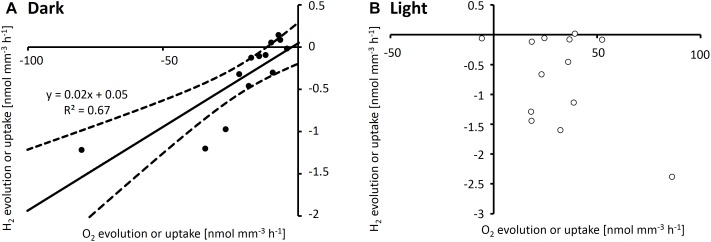
H_2_ and O_2_ evolution rates measured simultaneously by using two electrodes on the same *Trichodesmium* colonies in dark **(A)** and light **(B)**. All measurements were conducted after 2 pm. Note that all data shown are net rates, with positive numbers reflecting net evolution of H_2_ or O_2_ by a specific colony, and negative numbers reflecting net uptake of H_2_ or O_2_, respectively. **(A)** shows a linear regression (solid line) and 95% confidence interval of the fit (dashed lines).

Simultaneous measurements of H_2_ and O_2_ gradients performed by using two microelectrodes on the same colony showed a positive correlation (*R*^2^ = 0.67) between net O_2_ and H_2_ uptake when measured in the afternoon in the dark, with a ratio of ca. 50 mol O_2_ per mol H_2_ taken up (Figure 4A). In light, when photosynthesis was active, O_2_ fluxes were not strongly correlated with H_2_ uptake (*R*^2^ = 0.2; Figure 4B).

### H_2_ Dynamics Over the Diel Cycle – Reaction Kinetics

**FIGURE 5:**
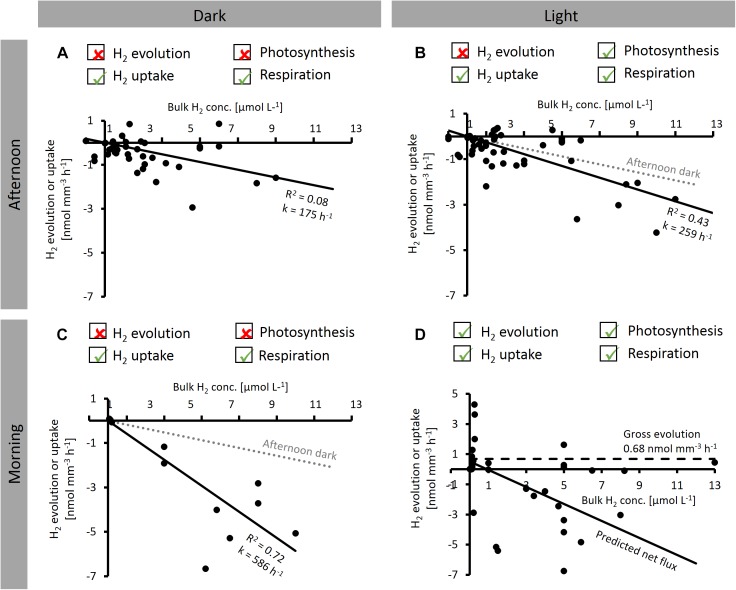
Dependence of net H_2_ evolution and uptake by *Trichodesmium* colonies on bulk H_2_ concentration measured at different times of the day [before 2 pm (morning) and after 2 pm (afternoon)] and under different light conditions. Ticked boxes above the plots indicate the dominant H_2_ and O_2_ producing and consuming processes under each condition, as indicated by microsensor results from this study. **(A–C)** show linear regression lines (*y*-axis intercept forced to zero). **(D)** Solid line shows predicted net H_2_ flux, dashed line shows gross H_2_ evolution, predicted as indicated in the text. Note that all data shown are net rates with positive numbers reflecting net evolution of H_2_ by a specific colony, and negative numbers reflecting net uptake of H_2_.

Aiming for a more systematic understanding of the kinetics of H_2_ uptake and evolution in *Trichodesmium*, we analyzed the dependence of H_2_ fluxes across the colony surface on the concentration of added H_2_ under different conditions (Figure 5). This approach of measuring H_2_ evolution under different H_2_ concentrations as well as light conditions over the diel cycle allowed us to explore the regulation of uptake hydrogenase activity on different levels, separating the roles of substrate availability, instantaneous light intensity and the underlying diel rhythm. To evaluate the relative importance of each of these parameters for H_2_ uptake, we firstly focused on H_2_ fluxes under conditions when we expected no H_2_ evolution, i.e., measurements carried out in the dark and in the afternoon, when nitrogenase is presumably inactive (Figure 5A–C). Under these conditions, the rate of H_2_ uptake can be simply described as

(5) $${\text{H}}_{2}\text{ uptake}=\text{k}\times [{\text{H}}_{2}],$$

where k is a pseudo first order kinetic rate constant with units of h^−1^, which appears as the slope of the regression lines in Figure 5A–C.

In the afternoon, H_2_ uptake was not significantly different between light and dark (*F*-test, *p* > 0.05; Figure 5A,B), with k values of 175 and 259 h^−1^ for dark and light, respectively. On the other hand, comparison of the dark k value determined in the afternoon to that determined in the morning shows that H_2_ uptake was strongly affected by the time of the day, with a threefold stimulation in the morning (*k* = 586 h^−1^; *F*-test, *p* < 0.05; Figure 5C).

To estimate the implications of our findings for H_2_ fluxes under natural conditions, we furthermore used our experimental data to predict the average H_2_ flux under typical bulk H_2_ concentrations in natural systems. Under N_2_-fixing conditions, when H_2_ evolution (by nitrogenase) and H_2_ uptake (by hydrogenase) co-occur (Figure 5D), the net H_2_ flux can either be positive (net evolution) or negative (net uptake), depending on the bulk H_2_ concentration. The rate of net H_2_ evolution under these conditions can be described as the difference between gross H_2_ evolution and H_2_ uptake, which is, in turn, given by the product of k and [H_2_] (cf. Eq. 5):

(6) $$\mathrm{Net}{\text{H}}_{2}\;\mathrm{evolution}=\mathrm{Gross}{\text{H}}_{2}\;\mathrm{evolution}-\text{k}\times [{\text{H}}_{2}]$$

Given that H_2_ uptake across the colony surface cannot persist when there is no H_2_ present in the bulk seawater, the rate of gross H_2_ evolution was estimated from net H_2_ evolution rates measured at the lowest bulk H_2_ concentrations in our experiments (average net H_2_ evolution below 0.5 μmol L^−1^ bulk H_2_). Please note that this concept of “gross H_2_ evolution” refers to fluxes across the colony surface, whereas significantly larger fluxes may occur *within* the colony. Assuming that nitrogenase activity is not dependent on external H_2_ concentrations, the gross H_2_ evolution rate would be constant across all H_2_ concentrations at 0.68 nmol mm^−3^ h^−1^ (dashed line in Figure 5D). Since light had only a minor effect on k (Figure 5B), we assumed k determined in the morning in dark to be valid also under light conditions. The resulting predicted net H_2_ flux is plotted in Figure 5D (solid line). Based on the *x*-axis intercept of this line, our data suggest net H_2_ evolution to occur below a bulk H_2_ concentration of ca. 1 μmol L^−1^, whereas at higher H_2_ concentrations, H_2_ uptake is dominant.

### Response of Mineral Iron Uptake to H_2_ Addition

**FIGURE 6:**
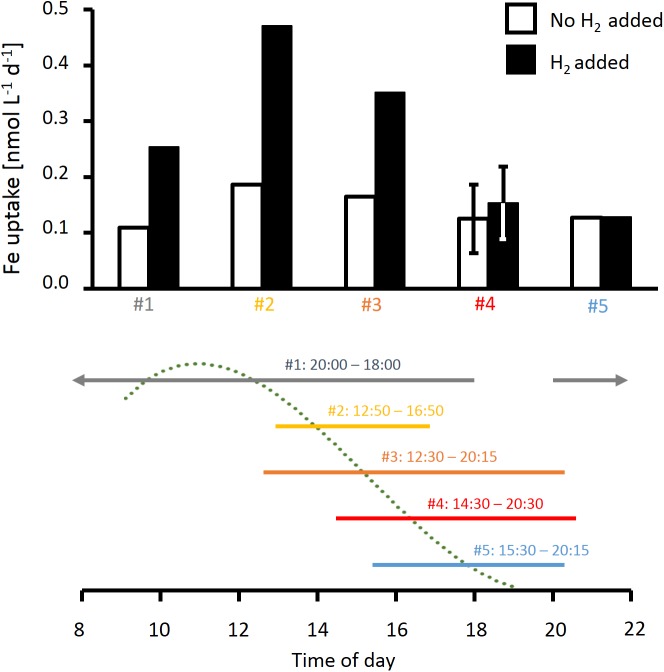
Effect of H_2_ on rates of iron uptake from ferrihydrite by natural *Trichodesmium* colonies measured at different times of the day. The time line below the plot indicates timing of the respective incubations as well as a typical diel cycle in N_2_ fixation (dotted line), which was obtained by a third order polynomial fit to N_2_ fixation rates measured in previous studies as specified in the legend of Figure 2. Error bars for incubation #4 show standard deviation of two replicate samples. Note incubation #1 continued through the night.

Mineral iron uptake by *Trichodesmium* colonies in incubations without added H_2_ was similar across five experiments conducted at different times of the day (Figure 6), with average uptake rates of 14 ± 3 fmol Fe colony^−1^ day^−1^. In the presence of H_2_, mineral iron uptake was strongly elevated in three out of five experiments, including both puff-shaped colonies (experiments #2 and #3; Figure 6) and tuft-shaped colonies (experiment #1; Figure 6). Over a whole day-night cycle, iron uptake was increased by more than a factor of 2 in the presence of H_2_ (experiment #1, Figure 6). In shorter incubations, the response in uptake to H_2_ was dependent on the time of the day. The strongest response was observed in incubations that were started at midday (experiments #2 and 3, Figure 6), and thus included the previously reported maximum in N_2_ fixation rates. In incubations lasting from the end of the N_2_ fixation period till the evening (experiments #4 and 5, Figure 6), there was no response to H_2_.

## Discussion

### H_2_ Uptake and Evolution by Individual Colonies

Our microsensor measurements of H_2_ and O_2_ gradients in single, field-collected *Trichodesmium* colonies demonstrate significant variability in H_2_ metabolism between individual colonies, which resulted in net H_2_ fluxes directed either in or out of the colony. Single *Trichodesmium* colonies thus provide distinct and highly diverse microenvironments with regard to H_2_ concentrations. Our experimental approach does not distinguish between the contribution of *Trichodesmium* and its associated bacteria to the H_2_ fluxes. However, the fact that *Trichodesmium* colonies in our study were always oxic (>100 μmol L^−1^ O_2_) precludes activity of many of the known H_2_-consuming taxa, such as anoxygenic phototrophs and sulfate reducing bacteria, and H_2_-producers such as fermenting cyanobacteria. It should be noted that gross H_2_ fluxes may be significantly larger than the net H_2_ fluxes determined in this study, particularly in the confined space of a colony microenvironment with close spatial coupling of sources and sinks.

Previous studies have reported a wide range of H_2_ evolution rates by *Trichodesmium*. Field studies in different locations across the North Atlantic and Caribbean showed H_2_ evolution rates ranging between 0.002 and 0.05 nmol colony^−1^ h^−1^ (Scranton, 1983, 1984; Scranton et al., 1987). The maximum net H_2_ evolution rate per colony in our study (0.08 nmol colony^−1^ h^−1^) strongly exceeds these previous field-based estimates. For laboratory cultures grown in single filaments under ca. 50 μmol photons m^−2^ s^−1^, net H_2_ evolution rates in the order of 1 nmol (μg chl *a*)^−1^h^−1^ and up to 3 nmol (μg chl *a*)^−1^h^−1^ were reported (Wilson et al., 2010, 2012). Using a chlorophyll *a* content per colony determined previously in the North Pacific Subtropical Gyre (14 ng colony^−1^; Eichner et al., 2017) to normalize H_2_ fluxes, our maximum estimate also exceeds net H_2_ evolution determined in the laboratory by approx. a factor of two (Wilson et al., 2010). Similarly, maximum net H_2_ uptake in our study [9 nmol H_2_ (μg chl *a*)^−1^ h^−1^] exceeds previous estimates of H_2_ uptake for *Trichodesmium* (estimated as the effect of the hydrogenase inhibitor carbon monoxide on net H_2_ evolution; Saino and Hattori, 1982) by approx. a factor of two. Aside from potential effects of experimental conditions among these studies, these comparisons demonstrate that H_2_ fluxes in and out of the microenvironment of single colonies can be significantly larger (at their diel maximum) than average H_2_ fluxes determined in incubations of several colonies or homogenous cultures.

In contrast to the previous reports of *net H*_2_ *evolution*, the average across all our measurements in light was a *net H*_2_ *uptake* rate of 0.01 ± 0.03 nmol colony^−1^ h^−1^. While experimental conditions in previous laboratory as well as field studies (such as different light intensities, colony formation and different species) most probably contribute to the differences between ours and previous results, possibly the most important difference was that our measurements were performed under micromolar H_2_ concentrations, while in natural waters H_2_ concentrations are typically in the low nanomolar range (e.g., Scranton, 1983; Wilson et al., 2013). Assuming a linear relation between net H_2_ evolution/uptake and bulk H_2_ concentration (Figure 5D), we extrapolated our data to lower H_2_ concentrations, and predicted that under bulk H_2_ concentrations below ca. 1 μmol L^−1^, H_2_ evolution would exceed H_2_ uptake. Under natural H_2_ conditions, the net H_2_ flux would thus – on average – be directed out of the colony, implying net H_2_ evolution in line with previous observations. However, our measurements also demonstrate that substantial variability in H_2_ fluxes (as observed within and between previous studies) can even be manifested on the level of single colonies sampled over a short period of time at the same location.

### Dependence of H_2_ Fluxes on Light and the Diel Cycle

Measuring the steady-state H_2_ gradients on the colony surface against a background of added H_2_ enabled us to estimate the capacity of *Trichodesmium* colonies for H_2_ uptake under various conditions. Since the H_2_ uptake rates were measured under artificially elevated background concentrations of H_2_, the rates we report should be considered as potential rates. The capacity for H_2_ uptake was not uniform over the diel cycle and dependent on light (Figure 2, 3), indicating that uptake hydrogenase is not only regulated by the availability of its substrate H_2_, in line with previous findings (Tamagnini et al., 2002). The fact that both net evolution and net uptake of H_2_ were observed reflects the delicate balance between the two highly active, opposing processes of H_2_ evolution by nitrogenase and H_2_ uptake by uptake hydrogenase. However, considering only conditions where nitrogenase is presumably inactive (afternoon and/or dark) allowed us to separate these process and indicated that light itself had only a minor effect on uptake hydrogenase (Figure 5A–C). Consequently, the light effects on net H_2_ fluxes in our study (Figure 2, 3) were most likely driven by the light response of nitrogenase activity, whereas uptake hydrogenase was not directly affected by light. The strong stimulation of dark H_2_ uptake in the morning compared to the afternoon (Figure 5C), in turn, indicates that H_2_ uptake is regulated over the diel cycle, potentially via an endogenous rhythm. The links between H_2_ metabolism, respiratory and photosynthetic electron transport in cyanobacteria make for a complex regulatory system. O_2_ fluxes measured at the same time and/or on the same colonies thus provided an important reference for gaining insights into the physiological mechanisms driving the H_2_ dynamics we observed in *Trichodesmium* colonies. In the following paragraph, we discuss these potential physiological mechanisms.

### Underlying Physiological Mechanisms

#### Processes Involved in the Dark

In the dark, O_2_ uptake by respiration and H_2_ uptake by hydrogenase were correlated (Figure 4A), in line with previously suggested links between these processes (e.g., Appel and Schulz, 1998). Uptake hydrogenases have been reported to be located on either the thylakoid membrane or the cytoplasmic membrane and to feed electrons into the plastoquinone (PQ) pool of the respiratory electron transport chain (ETC), potentially via a cytochrome-like anchoring unit that binds the H_2_-oxidizing subunit to the membrane and facilitates electron transport to the respiratory ETC (Appel and Schulz, 1998; Khanna et al., 2016). Electrons originating from H_2_ could then be transported from PQ to cytochrome b_6_f, and further to a terminal oxidase where they are used to reduce O_2_. The ratio of O_2_ to H_2_ uptake observed in the dark in the afternoon (Figure 4A) suggests that, under these conditions, 1 out of 50 electrons reducing O_2_ comes from H_2_, with the remainder originating from carbohydrates.

#### Processes Involved in the Light

In the light, O_2_ and H_2_ fluxes are determined by photosynthesis and N_2_ fixation in addition to respiration and H_2_ uptake. Net H_2_ evolution was observed in light but not in dark (Figure 2), which we attribute to the light-dependence of nitrogenase activity observed in previous studies on *Trichodesmium* (e.g., Rabouille et al., 2006; Staal et al., 2007). Activation of nitrogenase by light was also reflected in the instantaneous increase in H_2_ concentrations when the light was switched on during the time of highest N_2_ fixation (Figure 3). These findings are in agreement with previous studies showing instantaneous decreases in net H_2_ evolution in response to addition of NH_4_^+^ (which inhibits nitrogenase), DCMU (an inhibitor of PSII), and darkness in *Trichodesmium* (Wilson et al., 2010, 2012), as well as a direct dependence of H_2_ evolution on PSI activity in *Cyanothece* (Min and Sherman, 2010; Skizim et al., 2012). Notably, the interaction between O_2_ evolution, nitrogenase activity and H_2_ uptake can be modulated by the reduction state of the photosynthetic electron transport chain (Wilson et al., 2012), suggesting that the quantitative relationship between these processes is strongly dependent on the instantaneous light intensity. Due to the O_2_-sensitivity of uptake hydrogenase (e.g., Arp and Burris, 1981; Houchins and Burris, 1981; Axelsson and Lindblad, 2002), the high levels of photosynthetic O_2_ evolution in the afternoon (Figure 2C) might impede not only H_2_ evolution by nitrogenase but also H_2_ uptake, leading to lower levels of H_2_ uptake in the afternoon than in the morning (Figure 2B), and causing a loss of the correlation between H_2_ and O_2_ fluxes in the light (Figure 4B).

#### Regulation Over the Diel Cycle

The timing of highest variability in net H_2_ fluxes coincided with the time of highest net O_2_ uptake as well as the midday peak in N_2_ fixation reported previously (e.g., Berman-Frank et al., 2001). The down-regulation in net O_2_ evolution during this part of the day is believed to act as a mechanism to protect nitrogenase from O_2_ (e.g., Berman-Frank et al., 2001) and might additionally benefit uptake hydrogenase, which is also O_2_-sensitive (e.g., Houchins and Burris, 1981; Axelsson and Lindblad, 2002; Tamagnini et al., 2007). H_2_ evolution and N_2_ fixation rates have been previously shown to be correlated over a range of conditions (Tamagnini et al., 2002 and references therein), yet most previous studies on H_2_ evolution by *Trichodesmium* did not resolve H_2_ fluxes over the diel cycle. A laboratory study on *Trichodesmium* cultures grown in single filaments under a 12:12 h light-dark cycle at significantly lower intensities (44 μmol photons m^−2^ s^−1^) did not show the same diel pattern in net H_2_ evolution (Wilson et al., 2010), potentially due to feedbacks of colony formation and/or the range of light intensities in our field study.

During the morning, when N_2_ fixation and thus H_2_ evolution were presumably highest, elevated H_2_ concentrations might elicit an increase in the activity of uptake hydrogenase. The fact that we observed high *net* H_2_ evolution as well as uptake at this time of the day may reflect a mismatch in timing between the activation of nitrogenase and uptake hydrogenase – a slight offset in timing would result in strong net fluxes, while a balanced system with a constant fraction of H_2_ recycling would result in minimal net fluxes. Interestingly, H_2_ uptake was highest at morning/midday even in dark measurements (Figure 2B) where nitrogenase was presumably inactive, suggesting that uptake hydrogenase activity is regulated not only by H_2_ availability (i.e., by nitrogenase activity) or by light, but potentially also via an endogenous rhythm, as shown for nitrogenase in *Trichodesmium* (Chen et al., 1998). Both, uptake hydrogenases and nitrogen metabolism, are regulated by the transcriptional regulator NtcA (e.g., Tamagnini et al., 2002; Khanna et al., 2016), which was suggested to be under the control of a circadian rhythm in *Cyanothece* (Bradley and Reddy, 1997). In the afternoon, H_2_ fluxes were less pronounced (Figure 2A,B) and did not respond to light (Figure 3B) as strongly as in the morning, even though colonies were clearly active and generally responsive to light as indicated by the high O_2_ evolution in the light (Figure 2C, 3C). H_2_ evolution by nitrogenase can presumably not respond to light at this time of the day since nitrogenase becomes inactivated by post-translational modification in the afternoon (Zehr et al., 1993). The fact that also H_2_ uptake did not respond strongly to light (Figure 3) and uptake rates were low despite addition of H_2_ (Figure 2) at this time of the day suggests that uptake hydrogenase might be down-regulated in the afternoon in a similar way as nitrogenase.

### The Potential Role of H_2_ in Iron Acquisition

**FIGURE 7:**
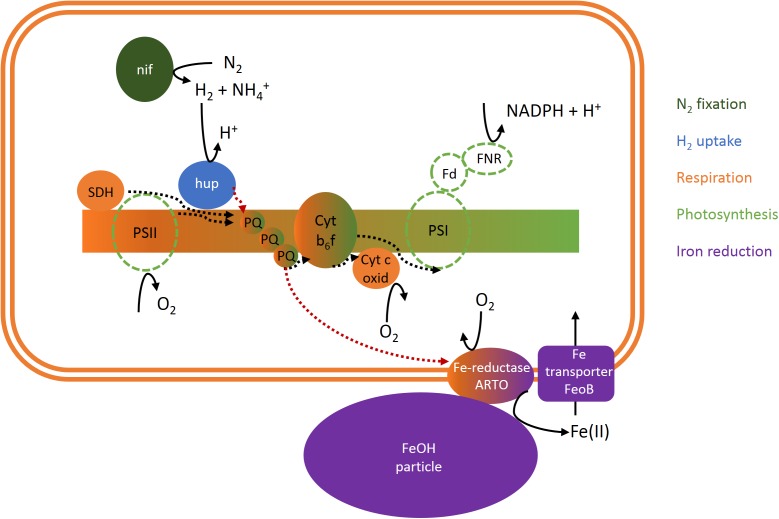
Scheme of a potential cellular link between N_2_ fixation, H_2_ and O_2_ fluxes and iron uptake in *Trichodesmium.* Dotted lines indicate electron flow. Please note that uptake hydrogenase may be restricted to non-photosynthetic diazocytes and/or located on the cytoplasmic membrane instead of the thylakoid membrane and thus coupled to respiratory electron transport but not photosynthesis (Tamagnini et al., 2002; Khanna et al., 2016). ARTO, alternative respiratory terminal oxidase; Cyt b_6_f, cytochrome b_6_f complex; Cyt c oxid, cytochrome c oxidase; Fd, ferredoxin; FeoB, ferrous iron transporter B; FeOH, iron oxide; FNR, ferredoxin-NADPH-oxidoreductase; hup, uptake hydrogenase; nif, nitrogenase; PQ, plastoquinone; PSI, photosystem I; PSII, photosystem II; SDH, succinate dehydrogenase.

Using a recently optimized radiotracer method for measuring iron uptake from mineral iron by *Trichodesmium* colonies (Basu and Shaked, 2018), we were able to investigate the effects of H_2_ on iron uptake under conditions that closely resembled the acquisition of iron from dust under natural conditions. Interestingly, these experiments revealed that the uptake of mineral iron was strongly stimulated by the presence of H_2_ (Figure 6). This effect was observed across different colony morphologies, which are generally assumed to represent different species (e.g., Hynes et al., 2012). Also the timing of H_2_ uptake and iron uptake was synchronized: Both H_2_ uptake and evolution as well as the response of iron uptake to H_2_ were strongest around midday, whereas in the afternoon, both H_2_ uptake and iron uptake responded less strongly to H_2_. Taken together, these findings suggest a potential mechanistic link between iron uptake and H_2_ metabolism. While the exact mechanisms of mineral iron acquisition by *Trichodesmium* are yet to be elucidated, there are several indications for a reductive step involved in the process, such as the slow-down of iron uptake from ferrihydrite in the presence of the Fe(II) ligand ferrozine (Supplementary Figure 1). The electron source for this reduction has not been identified. Based on the large H_2_ fluxes in colonies in combination with the stimulation of iron uptake by H_2_ and indications for co-regulation of uptake hydrogenase and iron uptake over the diel cycle, we propose that H_2_ may function as an electron source for mineral iron reduction. While the quantitative importance of this potential mechanism needs to be investigated in future studies under H_2_ concentrations that more closely mimic natural conditions, we focus here on identifying a potential pathway of electron flow that might provide such a link between H_2_ and mineral iron reduction on the cellular level (Figure 7).

The link between uptake hydrogenase and the respiratory electron transport chain has been reported previously (e.g., Appel and Schulz, 1998; Wilson et al., 2010), and was reflected in the correlation between H_2_ and O_2_ uptake in our study (Figure 4). In *Synechocystis*, mineral iron reduction was recently suggested to be facilitated by the alternative respiratory terminal oxidase ARTO (Kranzler et al., 2014). ARTO is also expressed and regulated in response to iron availability in *Trichodesmium* (Polyviou et al., 2018). As it accepts electrons from PQ (Lea-Smith et al., 2016), it could potentially serve as a link between the respiratory electron transport chain and iron reduction. Since on average, cellular iron uptake rates are several orders of magnitude lower than respiration rates (e.g., iron uptake of ca. 1–9 × 10^−18^ mol Fe cell^−1^ d^−1^, Basu and Shaked, 2018, as compared to respiration of ca. 3 × 10^−12^ mol O_2_ cell^−1^ d^−1^, Eichner et al., 2017), only a small fraction of electrons in the respiratory pathway would need to be channeled to iron reduction to meet cellular iron demands, while the remaining electrons would reduce O_2_. Nevertheless, a coupling of H_2_ uptake to iron acquisition via the respiratory ETC (i.e., electron flow from uptake hydrogenase via the PQ pool to ARTO) could explain the stimulation of iron uptake by H_2_ observed in this study (Figure 6) during the time of the day when high rates of H_2_ uptake were observed (Figure 2, 3). The reduced activity of uptake hydrogenase in the afternoon/evening (Figure 2, 3), potentially caused by a down-regulation/post-translational modification of the protein, in turn, could explain the lack of a response in iron uptake to H_2_ at this time of the day (Figure 6).

Previous studies on heterocystous, filamentous cyanobacteria suggested that uptake hydrogenase is present only in heterocysts (e.g., Tamagnini et al., 2007). Such a restriction of uptake hydrogenase to N_2_-fixing cells would provide not only a tight spatial coupling between H_2_ evolution and uptake, but also the benefit of reduced O_2_ concentrations that would protect both nitrogenase and uptake hydrogenase from oxidative damage. In *Trichodesmium*, it has been suggested that only 10–20% of cells in each filament, the diazocytes, express nitrogenase and actively fix N_2_ (e.g., Bergman and Carpenter, 1991, Berman-Frank et al., 2001). Notably, those cells would have a significantly higher iron requirement associated to synthesis of the iron-rich nitrogenase than vegetative cells (19–53% of cellular iron was estimated to be bound in nitrogenase on average across all cell types; Kustka et al., 2003). The pathway of iron reduction coupled to H_2_ uptake we propose here could provide a mechanism of surplus iron reduction to fuel the enhanced iron demand in these cells. The restriction of hydrogenase to diazocytes would also imply that H_2_ uptake and photosynthesis are separated on a single-cell level, and thus do not share the same electron transport chain, potentially explaining why light did not have strong direct effects on H_2_ uptake (at the time when nitrogenase was presumably inactive; Figure 5). Furthermore, assuming that the H_2_ fluxes we detected as an average of all cells in a single colony were actually catalyzed by only 10–20% of the cells, it should be noted that the ratio of H_2_:O_2_ uptake in those cells would be 5–10× larger than the average ratio we measured (Figure 4). Particularly since photosynthetic water splitting is presumably not available as an electron source in diazocytes, H_2_ as an alternative electron source might be relatively more important in these cells. In summary, although the contribution of H_2_ to total electron flow in the ETC (as estimated from the ratio of H_2_:O_2_ uptake; Figure 4) was relatively low when averaged over all cells and determined outside of the reported peak in N_2_ fixation, we suggest that in specialized N_2_-fixing cells at the time of active N_2_ fixation, a significant part of the electron flux in the respiratory electron transport chain might be channeled from H_2_ to iron. Future studies should thus aim to confirm the physiological mechanism and quantitative importance of this pathway in *Trichodesmium*. Complementary molecular biological studies on the metabolic potential of associated bacteria in *Trichodesmium* colonies will bring further insights into the potential contribution of these bacteria to both hydrogen uptake and iron reduction within colonies.

## Conclusion

By high-resolution measurements of H_2_ and O_2_ gradients on single colonies, we were able to demonstrate highly variable H_2_ microenvironments in colonies of the important N_2_ fixer *Trichodesmium*. We found that the net H_2_ flux could be directed either in or out of the colony. H_2_ uptake was regulated not only by H_2_ availability and by light, but also over the diel cycle, potentially by an endogenous clock. Both H_2_ uptake and evolution were generally most active in the morning at the time of highest N_2_ fixation, reflecting the tight link between nitrogenase and uptake hydrogenase activity. The large variability in H_2_ fluxes between individual colonies analyzed under similar conditions furthermore highlights that single colonies may provide diverse micro-niches differing strongly in H_2_ metabolism, including systems characterized by either net H_2_ evolution or net H_2_ uptake. Given that the experimental approaches used in previous studies (measuring bulk H_2_ evolution rates under natural H_2_ concentrations) do not allow for detecting such potential hot-spots of net H_2_ uptake, our findings highlight the need for more broadly applying (1) methods that allow for quantifying H_2_ uptake rather than only net evolution (such as ^3^H_2_ assays; Wilson et al., 2013), and (2) high-resolution measurements that can reveal small-scale environmental variability (such as microsensors).

Combining H_2_ measurements with radiotracer experiments, we furthermore observed a strong stimulation of mineral iron uptake in the presence of H_2_, indicating a previously unrecognized link between H_2_ and acquisition of iron. We propose a potential pathway of electron flow linking N_2_ fixation, H_2_ uptake, respiration and iron reduction on the cellular level, which should be the subject of further physiological investigations. This link between H_2_ uptake and mineral iron acquisition adds a new component to the complex network of nutrient acquisition mechanisms employed by *Trichodesmium* in the colony microenvironment.

## Data Availability

The raw data supporting the conclusions of this manuscript will be made available by the authors, without undue reservation, to any qualified researcher.

## Author Contributions

ME, YS, and DB designed the study. ME performed the microsensor measurements with help by DB and analyzed the data. SB performed the iron uptake experiments and analyzed the data. ME drafted the manuscript. All authors contributed to the data interpretation and writing the manuscript, and read and approved the final version of the manuscript for submission.

## Conflict of Interest Statement

The authors declare that the research was conducted in the absence of any commercial or financial relationships that could be construed as a potential conflict of interest.

## Footnotes

1 http://genome.jgi-psf.org/finished_microbes/trier/trier.home.html
