# Supplementary material for: Hydrogen Dynamics in Trichodesmium Colonies and Their Potential Role in Mineral Iron Acquisition
Source: Front Microbiol. 2019 Jul 10;10:1565. doi: 10.3389/fmicb.2019.01565 (PMC6636555; doi:10.3389/fmicb.2019.01565)
Supplement: Supplementary file 1 [file Data_Sheet_1.docx]

Supplementary Material

# Supplementary Figures


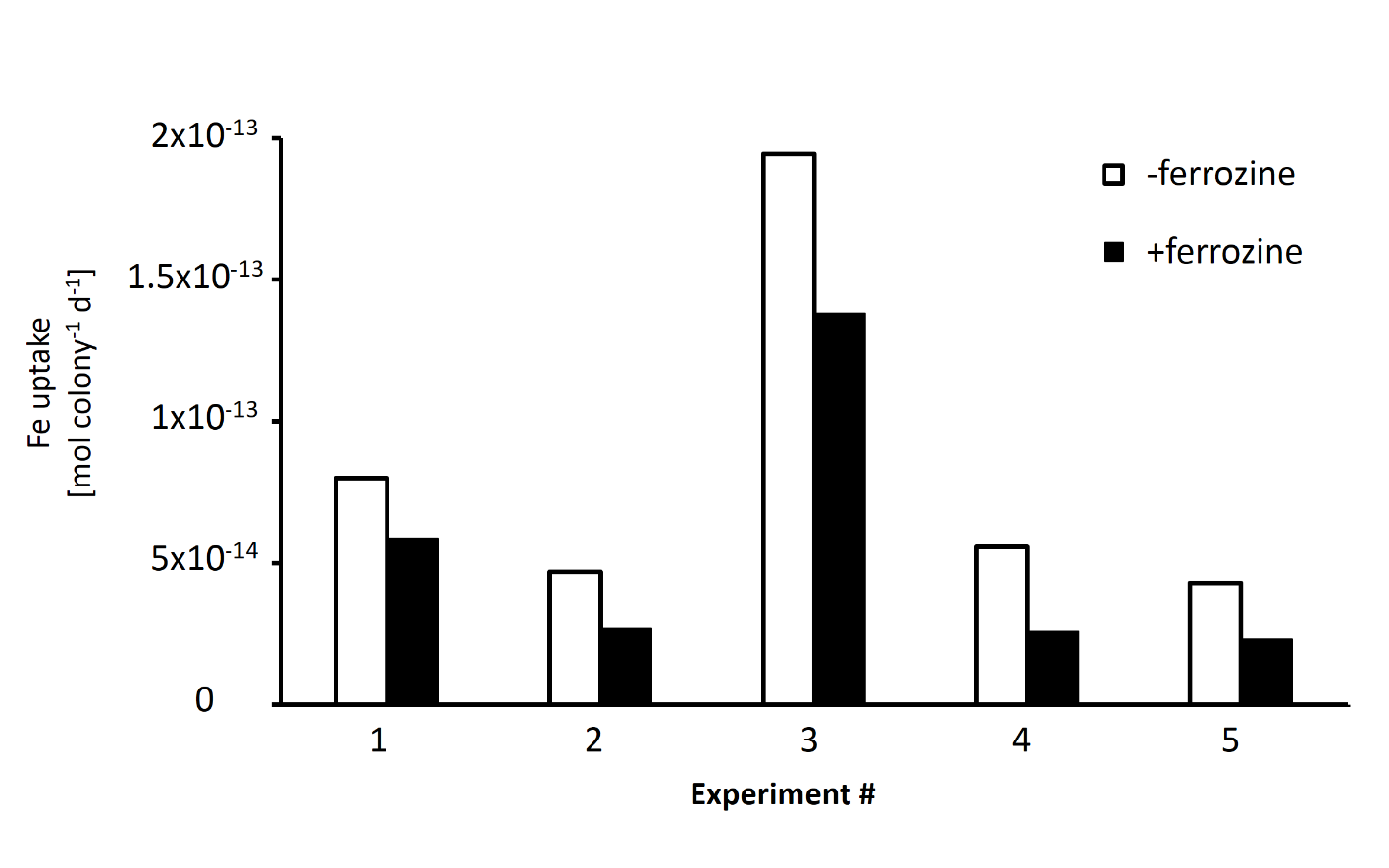


**Supplementary Figure 1:** Iron uptake from ferrihydrite colloids by *Trichodesmium* colonies measured in absence (open bars) and presence (300 µmol l^-1^; filled bars) of the Fe(II) ligand ferrozine. The inhibition of uptake by ferrozine is indicative of a reductive step during uptake. The 5 replicate experiments were performed on different dates between March 28^th^ and April 14^th^ 2015, on natural colonies collected in the Gulf of Eilat (20 to 30 colonies per sample).
